# Antibiotic-resistant *Escherichia coli* in deer and nearby water sources at Safari parks in Bangladesh

**DOI:** 10.14202/vetworld.2019.1578-1583

**Published:** 2019-10-19

**Authors:** Md Samun Sarker, Abdul Ahad, Saurav Kumar Ghosh, Md Shahriar Mannan, Arup Sen, Sirazul Islam, Md Bayzid, Zamila Bueaza Bupasha

**Affiliations:** 1 Department of Microbiology and Veterinary Public Health, Faculty of Veterinary Medicine, Chattogram Veterinary and Animal Sciences University, Chattogram 4225, Bangladesh; 2 Department of Livestock Services, Upazila Livestock Office, Thakurgaon Sadar, Thakurgaon, Bangladesh; 3 Department of Livestock Services, Upazila Livestock Office, Mithamain, Kishoreganj, Bangladesh; 4 Department of Pathology and Parasitology, Faculty of Veterinary Medicine, Chattogram Veterinary and Animal Sciences University, Chattogram 4225, Bangladesh.

**Keywords:** antibiotic-resistant, deer, *Escherichia coli*, lake, multidrug-resistant

## Abstract

**Background and Aim:**

The emergence and rapid dissemination of multidrug-resistant (MDR) bacteria in different ecosystems is a growing concern to human health, animal health, and the environment in recent years. The study aimed to determine the antibiotic resistance in *Escherichia coli* from deer and nearby water sources at two different Safari parks in Bangladesh.

**Materials and Methods:**

A number of 55 fresh fecal samples of deer and six water samples from nearby lakes were collected from two Safari parks. Samples were processed, cultured, and carried out biochemical tests for *E. coli*. The antibiotic susceptibility was determined by disk diffusion method. To identify the resistance genes, polymerase chain reaction was performed.

**Results:**

A total of 32 *E. coli* isolates from 55 fecal samples and 6 of 6 *E. coli* isolates from lake water were isolated. From fecal *E. coli* isolates, ampicillin and sulfamethoxazole were 90.63% (n=29/32) resistant and 87.5% (n=28/32) were resistant to tetracycline and nalidixic acid. High resistance was also observed to other antibiotics. On the contrary, all *E. coli* isolates from water sources were 100% (n=6/6) resistant to ampicillin, tetracycline, sulfamethoxazole, and nalidixic acid. MDR was revealed in all water samples, whereas 96.88% (n=31/32) was found in fecal isolates. A number of *bla*_TEM_, *tet* A, and *Sul*2 genes were detected from both isolates.

**Conclusion:**

This study for the 1^st^ time highlights, a significant proportion of *E. coli* isolates in wildlife deer and nearby water sources were MDR in Bangladesh.

## Introduction

Antibiotic resistance in wildlife is significant for human health due to the escalating of zoonotic diseases’ importance and predicting the emergence and dissemination of resistant pathogens among different ecosystems [1]. As major reservoirs, the domestic animals are usually considered for antibiotic-resistant bacteria. In recent times, interest in resistant bacteria and corresponding resistant genes isolated from wildlife and the environment has increased. It is evident that resistant bacteria at different environmental compartments act as a vector and reservoirs due to widespread of multidrug-resistant (MDR) bacteria [2].

*Escherichia coli*, commensal bacteria of warm-blooded animals, is widely used as fecal contamination indicator [3]. The presence of pathogenic strains of *E. coli* in wild animals, such as deer, may comprise a risk for human and other animals’ health [4]. The occurrence of antibiotic resistance in *E. coli* isolated from wild animals is not directly exposed to antibiotic treatment [5,6]. Through the attainment of resistant bacteria from human settings, agriculture, pharmaceuticals, hospitals, and associated tainted environments, they may be infected with antibiotic-resistant bacteria [7]. Even, in the pristine environments, where there was no direct human influence such as habitation, hospitals, and agricultural farming, resistant bacteria were reported [8,9].

There are a large number of captive deer at two different Safari parks in Bangladesh. Antimicrobials are not administered except for severe injury or disease outbreak. The purpose of raised deer at Safari parks is to conserve and for recreation of visitors. Nearby of deer habitat in Safari parks have a number of lakes that are used as the main source of drinking water of deer, easily polluted by visitors, droppings of wild birds and farming. The occurrence of antibiotic-resistant *E. coli* in wildlife and their environments have been reported worldwide [10-13]. Antibiotic-resistant *E. coli* has been reported in Bengal tigers at Safari parks in Bangladesh [14]. However, to the best of our knowledge, no previous research has been focused on antibiotic-resistant in *E. coli* isolated from deer and surrounding environments in Bangladesh.

This study aimed to assess the antibiotic-resistant with some corresponding resistant genes of *E. coli* in deer and nearby aquatic sources at two different Safari parks in Bangladesh.

## Materials and Methods

### Ethical approval

Since samples were collected from the environment, ethical approval was not necessary for this study.

### Sample collection

Environmental fresh fecal swab samples were collected aseptically from deer from two different Safari parks in Bangladesh, namely, Bangabandhu Sheikh Mujib Safari Park, Gazipur (latitude 23.71 and longitude 90.42, n=30) and Bangabandhu Sheikh Mujib Safari Park, Cox’s Bazar (latitude 21.43 and longitude 92.01, n=25), during the period of January-March 2016. It is interesting to note that the name of both Safari parks are same but they differ only in location. Immediately after collection, all samples were stored into a sterile screw-capped Falcon tube containing buffered peptone water (BPW). Along with feces, three water samples were also collected from each Safari park’s lake during that time. In a sterile Falcon tube, 40 ml of water sample was taken. Fecal and water samples were kept into an icebox and shipped in an unbroken freeze chain to the Poultry Research and Training Centre (PRTC), Chattogram Veterinary and Animal Sciences University (CVASU) as early as possible.

### *E. coli* isolation and identification

Fecal sample in BPW (Oxoid, UK) was incubated overnight at 37°C for enrichment. One loop full of enriched broth was plated onto MacConkey agar (Oxoid, UK) from BPW, incubated for 18-24 h at 37°C. 50 μl of water sample was directly inoculated onto MacConkey agar and incubated overnight at 37°C. On MacConkey agar, large pink-colored colonies were suspected as *E. coli*. A single isolated colony from MacConkey agar was subjected onto Eosin Methylene Blue (EMB; Merck, Mumbai) agar, characteristic green colonies with metallic sheen indicated as positive one. Different biochemical tests were conducted to confirm *E. coli* as earlier described by Gupta *et al*. [15]. Positive *E. coli* isolates were preserved in trypticase soy broth (TSB; Oxoid, UK) with 15% glycerol at −80°C until use.

### Antibiotic susceptibility testing

The disk diffusion method was performed on Mueller-Hinton agar (Oxoid, UK) plate to assess the antibiotic susceptibility of *E. coli* isolates according to the guidelines and recommendations of the Clinical and Laboratory Standards Institute (CLSI) [16]. The following 10 antibiotics that have both human and veterinary importance were tested: Ampicillin (10 µg), tetracycline (30 µg), ceftriaxone (30 µg), erythromycin (15 µg), chloramphenicol (30 µg), sulfamethoxazole (25 µg), nalidixic acid (30 µg), ciprofloxacin (5 µg), colistin (10 µg), and gentamicin (10 µg) (HiMedia, India). The susceptibility results were interpreted by the CLSI [16]. Isolate being resistant to at least three antimicrobial agents was defined as multidrug resistance [17].

### DNA extraction

DNA was extracted by the boiling method [18] with slight modifications. In 1.5 ml sterile Eppendorf tube, 2-3 fresh colonies were taken that contained 200 µl of deionized water and vortexed thoroughly. The Eppendorf tube was heated at 99°C for 15 min and centrifuged at 15,000 rpm for 2 min. The collected supernatant was used as a DNA template and stored at −20°C until use.

### Detection of antibiotic-resistant genes

Polymerase chain reaction (PCR) was performed to detect the antibiotic-resistant genes in a final volume of 25 μl consisting of 12.5 μl dream Taq PCR Master Mix (Thermo Scientific, USA), 0.5 μl of each primer, 1 μl template DNA, and 10.5 μl deionized water. Previously published PCR conditions were used to determine the presence of *bla*_TEM_, *tet* A, *tet* B, *tet* C, and *sul2* genes (Table-1) [19-22]. PCR was carried out using a thermocycler (2720 Thermal Cycler, Applied Biosystems, USA). The amplified products of PCR were then visualized on 1.5% agarose gels stained with ethidium bromide (0.5 μg/ml) (Sigma-Aldrich, USA). A DNA ladder (100-bp, Thermo Fisher Scientific) was used to determine the size of PCR products. The gel was inspected visually and photographed using an ultraviolet transilluminator (BDA digital, Biometra GmbH, Germany). As a positive control for a particular gene, previously isolated strain harboring of that gene was used from Microbiology Laboratory, CVASU.

**Table 1:** Primers used to identify antibiotic-resistant genes, *bla*_TEM_, *tet* A, *tet* B, *tet* C, and *sul*2.

Target gene	Primer sequence (5’- 3’)	Amplicon size (bp)	Annealing temperature (°C)	References
*bla*_TEM_	F: TACGATACGGGAGGGCTTAC R: TTCCTGTTTTTGCTCACCCA	716	53	[19]
*tet* A	F: GCTACATCCTGCTTGCCTTC R: CATAGATCGCCGTGAAGAGG	210	55	[20]
*tet* B	F: TTGGTTAGGGGCAAGTTTTG R: GTAATGGGCCAATAACACCG	659	55	[21]
*tet* C	F: CTTGAGAGCCTTCAACCCAG R: ATGGTCGTCATCTACCTGCC	418	55	[21]
*sul*2	F: CGGCATCGTCAACATAACCT R: TGTGCGGATGAAGTCAGCTC	721	66	[22]

### Statistical analysis

Data were managed into a spreadsheet program (Excel 2010, Microsoft Corporation). The proportion of samples with 95% confidence interval and other descriptive statistics were calculated by QuickCalcs GraphPad software.

## Results

### Proportion and cultural characteristics of *E. coli*

A proportion of 60% (n=18/30, 95% CI 42.29-75.44) *E. coli* from deer feces and 100% (n=3/3, 95% CI 38.25-100) *E. coli* from water samples was isolated from Bangabandhu Sheikh Mujib Safari Park, Gazipur, whereas 56% (n=14/25, 95% CI 37.05-73.35) and 100% (n=3/3, 95% CI 38.25-100) *E. coli* found in deer feces and water samples, respectively, at Bangabandhu Sheikh Mujib Safari Park, Cox’s Bazar. Overall, 58.18% (n=32/55, 95% CI 45.02-70.27) *E. coli* from feces and 100% (n=6/6, 95% CI 55.72-100) *E. coli* in water samples were isolated. Positive *E. coli* isolates produced bright pink colonies on MacConkey agar and characteristic green colonies with the metallic sheen on EMB agar. In methyl red and indole production test, all isolates were positive but negative to Voges–Proskauer test.

### Antibiotic resistance

**Figure-1:**
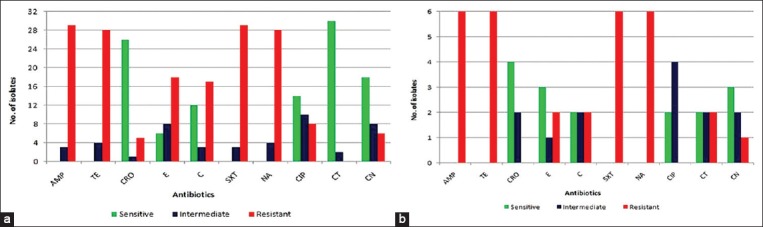
(a) Antibiogram profile of deer *Escherichia coli* isolates against different antibiotics (b) Antibiogram profile of water *E. coli* isolates against different antibiotics.

Of the 32 tested isolates of *E. coli* from feces, 90.63% (n=29/32) were resistant to ampicillin and sulfamethoxazole and 87.5% (n=28/32) were resistant to tetracycline and nalidixic acid. Resistance to erythromycin and chloramphenicol was 56.25% (n=18/32) and 53.13% (n=17/32), respectively. Results also dictated 93.75% (n=30/32) of *E. coli* isolates were sensitive to colistin followed by ceftriaxone (81.25%, n=26/32) and gentamicin (56.25%, n=18/32). On the contrary, all *E. coli* isolates were resistant to ampicillin, tetracycline, sulfamethoxazole, and nalidixic acid from water. None of the isolates were resistant to ceftriaxone and ciprofloxacin. Antibiotic susceptibility to different antibiotics is illustrated in Figure-1a and b. All of the *E. coli* isolates from deer unveiled that 96.88% (n=31/32) were MDR, whereas 100% (n=6/6) MDR were revealed in water samples. The common antibiotic-resistant patterns were ampicillin, tetracycline, sulfamethoxazole, and nalidixic acid for both feces and water isolates. There were 20 and four different MDR patterns were identified in fecal and water isolates, respectively (Table-2).

**Table 2:** Antibiotic-resistant patterns in *Escherichia coli* isolates from deer (n=32) and water (n=6).

Antibiotic-resistant patterns	Number of isolates	Source
AMP-TE	1	Feces
AMP-E-C	1	Feces
AMP-TE-SXT-NA	4	Feces
TE-CRO-SXT-NA	1	Feces
TE-E-C-CIP	1	Feces
AMP-TE-SXT-NA-CN	1	Feces
AMP-TET-E-C-SXT	1	Feces
AMP-TE-E-SXT-NA	4	Feces
AMP-E-SXT-NA-CN	1	Feces
AMP-TE-C-SXT-NA	1	Feces
AMP-E-SXT-NA-CIP	1	Feces
AMP-TE-E-C-SXT-NA	4	Feces
AMP-TE-CRO-E-SXT-NA	1	Feces
AMP-TE-C-SXT-NA-CN	2	Feces
TE-CRO-E-C-SXT-NA	1	Feces
AMP-C-SXT-NA-CIP-CN	1	Feces
AMP-TE-C-SXT-NA-CIP	2	Feces
AMP-TE-E-C-SXT-NA-CIP	2	Feces
AMP-TE-CRO-C-SXT-NA-CN	1	Feces
AMP-TE-CRO-E-SXT-NA-CIP	1	Feces
AMP-TE-SXT-NA	1	Water
AMP-TE-SXT-NA-CN	1	Water
AMP-TE-SXT-NA-CT	2	Water
AMP-TE-E-C-SXT-NA	2	Water

AMP=Ampicillin, TE=Tetracycline, CRO=Ceftriaxone, E=Erythromycin, C=Chloramphenicol, SXT=Sulfamethoxazole, NA=Nalidixic acid, CIP=Ciprofloxacin, CT=Colistin, CN=Gentamicin

### Antibiotic-resistant genes

We have characterized some of the targeting antibiotic-resistant genes on a molecular basis, namely, *bla*_TEM_ (β-lactamase resistant gene), *tet* A, *tet* B, *tet* C (tetracycline-resistant gene), and *sul2* (sulfur drug-resistant gene). In case of feces, of 29 ampicillin and sulfamethoxazole resistant isolates, 58.62% (n=17/29) and 34.48% (n=10/29) exposed *bla*_TEM_ and *sul2* genes, respectively. Eleven (39.29%) of the 28 tetracycline-resistant isolates contained the *tet* A gene. On the contrary, 66.67% (n=4/6) of water isolates contained *bla*_TEM_ gene, whereas 33.33% (n=2/6) of isolates carried both *tet* A and *sul2* gene. *tet* B and *tet* C gene were not exposed in any of the tested isolates from both sources.

## Discussion

In the present study, the overall proportion of fecal carriage *E. coli* from deer at two different Safari parks was 58.18%. Based on exhaustive literature searches, there are no available reports that predate our detection of *E. coli* in deer at Safari parks in Bangladesh. A similar study reported by Alonso *et al*. [23] who found 59% of *E. coli* from wild deer, whereas Carroll *et al*. [24] isolated 83% of *E. coli*. These variations may be due to the sample size, contamination of fecal samples with the surrounding environment, and geographical distribution.

Deer *E. coli* isolates were subjected to 10 antimicrobial agents to assess the antibiotic susceptibility pattern. *E. coli* isolates were resistant to at least two antibiotics, most commonly practiced in human and veterinary medicine in Bangladesh. In our study, the common phenotypic-resistant patterns were ampicillin, tetracycline, sulfamethoxazole, and nalidixic acid. We found that 90.63% (n=29/32) of resistance to ampicillin and sulfamethoxazole and 87.5% (n=28/32) of resistance to tetracycline and nalidixic acid. Higher resistance was also observed against erythromycin and chloramphenicol at 56.25% (n=18/32) and 53.13% (n=17/32), respectively. This resistance profile is higher than Li *et al*. [25] who reported that ampicillin, sulfamethazine, and tetracycline were 71.4%, 82.7%, and 80% resistant, respectively, in Northeastern China. However, 93.75% (n=30/32) of *E. coli* isolates were sensitive to colistin followed by ceftriaxone (81.25%) and gentamicin (56.25%). Resembling to our study, antibiotic-resistant *E. coli* were found in the environmental and biological sources such as human urine, human feces, sheep, goat, cattle, chicken, pigeon, broiler, duck, soil, and drain sewage in Bangladesh [26,27]. The results we have observed are a matter of great concern as to how these deer acquired resistance, though they were treated very rarely with these human-associated antibiotics at Safari parks. This may be due to deforestation and spillage of organism from human dwellings and wildlife in both directions.

From the nearby lakes, water samples were collected as lake water is supplied to deer as their drinking water. *E. coli* was isolated from all water samples, demonstrated the same resistance pattern as like deer isolates. About 100% of water isolates were resistant to ampicillin, tetracycline, sulfamethoxazole, and nalidixic acid. The studied area is in the main route of bird migration in Bangladesh and lake hosts thousands of wild birds. The wild birds can act as vectors and reservoirs of antibiotic-resistant bacteria that have veterinary and medical importance [28]. Wild birds pick up food from various environments and human surroundings are heavily polluted by resistant bacteria in Bangladesh [29] and lead to polluting natural water reservoirs such as lakes and rivers by inducing fecal contaminates [30] and dissemination of resistant bacteria. We have speculated that antibiotic resistance might have been transferred to deer through *E. coli* contaminated water. Moreover, in Bangladesh, animal manure is commonly used in agricultural application, which is a significant pathway into the terrestrial environment for the introduction of resistant bacteria. As a result, deer may acquire resistant bacteria during their grazing on pastureland.

Our study revealed that 96.88% of deer *E. coli* isolates were MDR, whereas 100% MDR was found in water isolates. Due to the indiscriminate victimization of antimicrobial agents, MDR strains may apparently occur with high incidence [31]. However, the findings of the MDR patterns of this study will help for the choice of drugs for the veterinarians to practice at Safari parks.

A number of different resistant genes, namely, *bla*_TEM_, *tet* A, and *sul2* were detected in both fecal and water *E. coli* in the present study. To the best of author’s knowledge, there are no previous reports on antibiotic-resistant with associated genes in *E. coli* from deer population at Safari parks in Bangladesh. The findings were in line with the data obtained from other countries by Alonso *et al*. [23] and Li *et al*. [25] who reported *bla*_TEM_, *tet* A, and *sul2* genes in *E. coli* isolates in wild deer. The spreading of resistance genes in natural environment may be a great threat to human and animal health.

## Conclusion

The study dictated the presence of MDR and some of their corresponding resistant genes in deer and nearby aquatic sources at Safari parks in Bangladesh. Nationwide effective antimicrobial resistance surveillance is badly needed to monitor the antimicrobials use and understanding the role of antimicrobial-resistant bacteria in the domestic and wild environments to diminish the public health challenge.

## Authors’ Contributions

MSS and AA conceived and designed the study. MSS and SKG collected samples from Safari parks. MSS, ZBB, and MB performed laboratory experiments. AS and MSM analyzed the data. MSS wrote the manuscript. AA and SI critically reviewed the manuscript. All the authors read and approved the manuscript for publication.
